# Evaluation of Cardiovascular Autonomic Nervous System in Essential Tremor and Tremor Dominant Parkinson’s Disease

**DOI:** 10.3390/brainsci14040313

**Published:** 2024-03-26

**Authors:** Jakub J. Malkiewicz, Joanna Siuda

**Affiliations:** Department of Neurology, Faculty of Medical Sciences in Katowice, Medical University of Silesia in Katowice, University Clinical Center Prof. K. Gibiński, 14 Medyków Street, 40-752 Katowice, Poland; jsiuda@sum.edu.pl

**Keywords:** Parkinson’s disease, tremor, essential tremor, autonomic dysfunction, autonomic nervous system, orthostatic hypotension, heart rate variability, SCOPA-AUT, dysautonomia, differential diagnosis

## Abstract

(1) Background: The differential diagnosis of essential tremor (ET) and tremor-dominant Parkinson’s disease (TDPD) can be challenging. Only a few studies have investigated the autonomic nervous system (ANS) in ET. However, some of these suggested that heart rate variability (HRV) might be useful in the differential diagnosis. (2) Methods: Demographic and clinical data, including medications and comorbidities, were collected from 15 TDPD patients, 19 ET patients, and 20 healthy controls. Assessment with the SCOPA-AUT questionnaire, 5 min HRV analysis in time and frequency domains, and evaluation of orthostatic hypotension (OH) with tilt test were performed. (3) Results: There were no significant differences between all groups on the SCOPA-AUT questionnaire. PD patients had OH more frequently and a larger drop in systolic blood pressure (SBP) during the tilt test than ET patients and controls. HRV was affected in PD, but not in ET and controls. Power in the low frequency band, the standard deviation of all normal RR intervals and SBP drop were potentially useful in differential diagnosis with AUCs of 0.83, 0.78, and 0.83, respectively. (4) Conclusions: Cardiovascular ANS dysfunction was present in TDPD, but not in ET and controls. HRV analysis and assessment of SBP drop may be potentially useful in the differential diagnosis of ET and TDPD.

## 1. Introduction

Essential tremor (ET) and Parkinson’s disease (PD) are the most common movement disorders, with a prevalence of 3.2:1000 and 1–2:1000 in the general population, respectively [1,2]. Differentiation of ET and PD can be challenging, and misdiagnoses are not uncommon [3]. Methods such as [123I] metaiodobenzylguanidine (123MIBG) scintigraphy, 123I-ioflupane SPECT (DAT-SPECT), and transcranial sonography can be useful for differential diagnosis [4,5,6]. However, 123MIBG scintigraphy and DAT-SPECT are expensive, and assessment of the substantia nigra with transcranial sonography strongly depends on the experience of the physician performing the procedure and the presence of a sufficient temporal window [5].

It is well-established that non-motor symptoms, including autonomic nervous system (ANS) dysfunction, are present in PD, with some such symptoms appearing prior to the onset of motor symptoms [7,8]. Patients with PD often experience abnormalities in gastrointestinal, urogenital, cardiovascular, and thermoregulatory functions, which become more prevalent and severe as the disease progresses [7,8]. They frequently present symptoms such as excessive salivation, dysphagia, impaired gastric emptying, constipation, hypothermia, excessive sweating or hypohidrosis, nocturia, and increased frequency and urgency of micturition [7,8]. Cardiovascular manifestations of ANS dysfunction in PD are orthostatic and postprandial hypotension (OH), supine hypertension, and diminished heart rate variability [7,8]. Non-motor symptoms might also be potentially associated with ET, including depression and other psychiatric problems, as well as cognitive dysfunction [1]. There have also been some studies which assessed ANS functions in essential tremor patients. A few of them did not find dysautonomia in ET [9,10,11,12], but there have also been works that have noted ANS abnormalities associated with the disease [13,14,15]. Subjective symptoms of genitourinary and cardiovascular dysfunction, assessed using the SCOPA-AUT questionnaire in ET patients, were noted by two studies [11,13], but one of them did not find a difference between ET and controls in objective tests [11]. Another study found that there was no difference between controls and ET patients, except for in sialorrhea [9]. In both studies comparing subjective symptoms of dysautonomia, ET patients had less severe problems than PD patients [9,13]. In contrast to PD, cardiovascular ANS functions assessed objectively in ET were preserved in most studies [10,11,12,14]. However, cardiovascular abnormalities in multiple tests were found by Nassar et al. [15]. Data from studies that assessed HRV in small and precisely selected groups of patients with PD and ET has revealed that HRV analysis might be the next effective method to differentiate the diseases [10,12].

This study aimed to compare ANS functions evaluated with the SCOPA-AUT questionnaire, HRV analysis, and assessment of OH in ET patients, tremor dominant Parkinson’s disease (TDPD) patients, and healthy controls, and to assess the usefulness of ANS assessment in differentiation of the diseases. The hypotheses of the study were that dysautonomia is absent in ET and present in TDPD, and that autonomic nervous system assessment is helpful in the differential diagnosis of ET and TDPD.

## 2. Materials and Methods

Patients with TDPD and ET or ET plus syndrome admitted to the Department of Neurology of the University Clinical Center of the Medical University of Silesia in Katowice, Poland between January 2021 and March 2023 who gave informed consent were qualified to participate in the study. The inclusion criteria was diagnosis of ET, ET plus syndrome, or PD according to current criteria [16,17]; for PD patients, specifically diagnosis of the TDPD subtype, according to the publication by Stebbins et al., was required [18]. The exclusion criteria were as follows: glomerular filtration rate of below 60 mL/min/1.73 m^2^ calculated with the MDRD formula; present or suspected liver cirrhosis; diabetes mellitus (DM), if patients had clinically known neurological complications of DM, had HbA1c > 6.5%, were on insulin therapy, or had been diagnosed ≥5 years earlier [19,20]; uncompensated thyroid dysfunction; severe respiratory disorders; heart diseases, if New York Heart Association Functional Classification > 1; non-sinus heart rhythm, heart block, and arrhythmias; presence of dementia; peripheral neuropathy; or significant electrolyte disturbances. All exclusion criteria were established to eliminate or reduce the effects of factors which could affect the ANS or its assessment. The control group was recruited from amongst the spouses and caregivers of patients, providing they did not meet any of the exclusion criteria and did not have movement disorders.

Clinical and demographic data were collected, including laboratory tests, comorbidities, prescribed medications, and clinical and neuropsychological assessments. The control group was not assessed by a neuropsychologist. Laboratory blood tests included: complete blood count, C-reactive protein, fasting glucose, glycated hemoglobin, alanine and aspartate transaminases, gamma-glutamyltransferase, bilirubin, sodium, potassium, chloride, creatinine, thyrotropic hormone and thyroid hormones, vitamin B12, folate, homocysteine, ceruloplasmin, and serum copper. Daily levodopa equivalent dose (LEDD) was calculated as proposed by Tomlinson et al. [21]. Information about used medications was also noted, especially drugs like anticholinergics, cholinergic agents, nitrates, central sympatholytic agents, antipsychotics, antidepressants, antihypertensive agents, α–adrenergic antagonists, and β-blockers and primidone, which could potentially interfere with ANS functions. The Hoehn-Yahr scale (HYs) and the Polish version of the MDS-UPDRS scale part three were used to assess severity of PD, and MDS-UPDRS parts two and three were utilized to determine PD subtype [18,22]. The tremor research group essential tremor rating assessment scale performance subscale (TETRAS) was used to assess ET severity [23]. All patients were assessed after 12 h wash out of dopamine replacement therapy (DRT). In the cases of all PD patients, and some ET patients, the levodopa challenge test was performed.

An autonomic function assessment was conducted following the methodology outlined in our previous study [24]. It included the Polish version of the SCOPA-AUT questionnaire, HRV analysis during a 5 min supine rest, and a 5 min tilt test [25]. The assessment of the ANS was conducted between 7 am and 12 pm at a room temperature of 20–25 °C. During autonomic tests, patients were required to abstain from DRT for at least 12 h and were also instructed to avoid beverages, alcohol, and nicotine on the day of the assessment. A 5 min ECG from lead II was recorded after a 10 min rest using the Biopac MP150 Acquisition System. Related AcqKnowledge software with a sampling rate of 1000 Hz and a band pass filter of 0.5–35 Hz was applied. The ARTiiFACT software was used to correct artifacts and ectopic beats and perform HRV analysis [26]. Artifacts and ectopic beats were detected through visual inspection of the data with the aid of software algorithms and corrected using cubic spline interpolation. The standard deviation of all normal RR intervals (SDNN) and root mean square of the successive differences between adjacent normal RR intervals (RMSSD) were used for HRV analysis in the time domain. The fast Fourier transform (FFT) technique was used for the spectral analysis of HRV. Powers in three frequency bands were calculated: high frequency (HF: 0.15–0.4 Hz); low frequency (LF: 0.04–0.15 Hz); and very low frequency (VLF: <0.04 Hz). LF/HF ratio was also obtained. HRV analysis assessment was performed in accordance with the Task Force of the European Society of Cardiology and the North American Society of Pacing and Electrophysiology [27]. After 15 min of supine rest, a 5 min head-up tilt test to a 60° angle was used to assess OH. Patients were diagnosed with OH if they experienced a sustained drop in systolic blood pressure (SBP) of ≥20 mmHg and/or diastolic blood pressure (DBP) of ≥10 mmHg within 5 min of the tilt test [28,29]. Changes in heart rate (HR) during the tilt test were also measured. The patients were diagnosed with neurogenic orthostatic hypotension (NOH) if their ΔHR/ΔSBP ratio was less than 0.5 bpm/mmHg, as per the publication by Norcliffe-Kaufmann et al. [30].

Statistical analysis was performed using STATISTICA v.13 PL software (Tibico Software Inc, Palo Alto, CA, USA). The quantitative variables were presented as an arithmetic mean (standard deviation) for normally distributed variables, or a median (interquartile range) for non-normally distributed variables. The Shapiro-Wilk test was used to assess the normality of distribution and the Brown–Forsythe test was used to check the homogeneity of variance. The U-Mann-Whitney test and Kruskal-Wallis ANOVA were used in cases of non-normally distributed variables. One-way ANOVA was utilized in cases of normally distributed variables, if the assumption of homogeneity of variance was not violated. The Tukey HSD test was used because of its intermediate risk of I-type error and statistical power in comparison to alternative tests. For the Kruskal-Wallis ANOVA, a post-hoc comparison test provided and recommended by Tibico software was used [31]. Qualitative variables were presented as percentages. To compare qualitative variables, a chi-square test and Fisher’s exact test were used. The Fisher’s exact or Chi-square tests with Bonferroni correction for multiple comparisons were used for post-hoc comparisons. Bonferroni correction for multiple comparisons was used due to the simplicity of its application in comparison to alternative methods. The cut-off point for *p*-values was set at 0.05, or, in cases where Bonferroni correction was used, 0.017. If there were significant differences in ANS parameters between TDPD and ET, sensitivity and specificity were calculated for ET diagnosis. For continuous variables, receiver operating characteristic (ROC) analysis was performed, ROC area under the curve (AUC) with confidence intervals (CI) was calculated, and cut points were established using the Youden index.

The study protocol was reviewed and approved by the Bioethical Ie of the Medical University of Silesia in Katowice (PCN/0222/KB1/27/I/20) and performed in accordance with the 1975 Declaration of Helsinki. Written informed consent was obtained from all subjects.

## 3. Results

A total of 19 ET patients, 15 TDPD patients, and 20 controls were included in the study. There were no statistically significant differences between groups in terms of age, sex, or comorbidities. The disease duration was longer for ET than PD (*p* = 0.015). None of the patients used anticholinergic or cholinergic agents, nitrates, central sympatholytic agents, first-generation antipsychotics, or antiarrhythmics other than β–blockers. Only one patient with PD used an atypical antipsychotic, quetiapine, at a dose of 25 mg per day. One PD patient took mirtazapine, and one ET patient used mianserin at a dose of 30 mg per day. There were no significant differences in the use of serotonin reuptake inhibitors (SSRIs) between the groups. Renin–angiotensin system antagonists (RAS) were more commonly used in ET patients in comparison with controls. There were no relevant differences in the use of other antihypertensive agents and α–adrenergic antagonists between groups. β–blockers were most commonly used in ET patients, but there was no statistically significant difference in a number of patients using β–blockers between groups. Propranolol was used in five patients with ET at a median dose of 60 (range 20–120) mg/d, metoprolol in two ET patients at doses of 50 and 100 mg/d, bisoprolol in two patients at doses of 3.75 and 2.5 mg/d, and betaxolol at a dose of 20 mg/d in one patient. In PD, two patients used propranolol at a dose of 60 mg/d, and another two used nebivolol at a dose of 5 mg/d. Four controls used metoprolol at doses of 12.5, 25, and in two cases 50 mg/d, respectively. Primidone was used by five ET patients at a median dose of 500 mg (range 125–1250) mg/d. Six ET patients were using dopaminergic drugs as they were referred to the clinic with a susception of PD, and the diagnosis was verified negatively. In two dubious cases, DAT-SPECT was performed and excluded PD. LEDD was significantly larger in TDPD patients in comparison with ET and controls. The details of demographic and clinical data are presented in Table 1.

There were not any significant differences between groups regarding subjective complaints of dysautonomia in the SCOPA-AUT, with the exception of the pupillomotor domain (*p* = 0.037), but significant differences for this domain were not found in post-hoc comparisons. TDPD patients had significantly lower HRV than ET and the control group for SDNN (TDPD vs. ET, *p* = 0.016; TDPD vs. control group, *p* < 0.001), VLF (TDPD vs. control group, *p* < 0.001), and LF bands (TDPD vs. ET, *p* = 0.008; TDPD vs. control group, *p* < 0.001). TDPD patients had larger SBP drops than ET patients (*p* < 0.001) and the control group (*p* < 0.001), but there was no statistically significant difference between groups in terms of DBP drops and ΔHR. TDPD patients more frequently had SBP drops ≥20 mmHg and NOH than other groups (TDPD vs. ET *p* = 0.011; TDPD vs. controls *p* = 0.009). OH was more common in TDPD vs. ET (0.011). However, there were no significant differences in the number of patients with DBP drop ≥10 mmHg, concomitant SBP drop ≥20 mmHg, and DBP drop ≥10 mmHg. Details of the results of ANS assessments are presented in Table 2.

**Figure 1:**
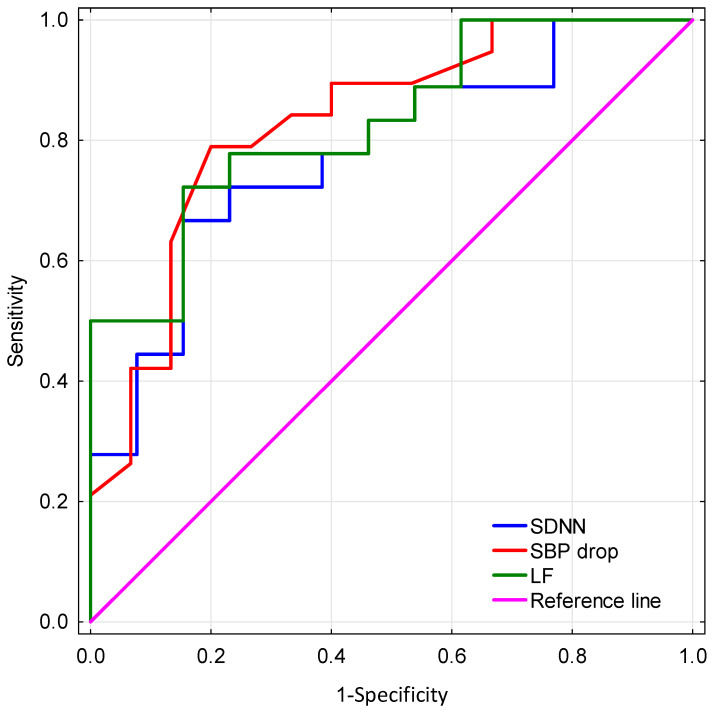
Comparison of receiver operating characteristic (ROC) curves for assessed parameters in differentiating the ET group from the TDPD group. SDNN—standard deviation of all normal RR intervals, SBP—systolic blood pressure, LF—power in low frequency band.

The results of the ROC analysis showed that the AUC values were 0.829 (CI 0.687–0.971), 0.778 (CI 0.614–0.943), and 0.83 (CI 0.668–0.971) for LF, SDNN, and SBP drop during tilt test, respectively. SBP drop sensitivity was 79% and specificity was 80% for a cut point of 3 mmHg. LF had a sensitivity of 72% and specificity of 85% for a cut point of 153 ms^2^. SDNN had a sensitivity of 67% and specificity of 85% for a cut point of 28 ms. ROC curves for assessed parameters in differentiating the ET group from the TDPD group are presented in Figure 1.

## 4. Discussion

Some previous studies found that PD patients had more severe dysautonomia assessed by the SCOPA-AUT questionnaire in comparison to ET [9,13]. Lee et al. also found that ET patients reported more subjective complaints than controls [13]. Symptoms of dysautonomia in PD tend to worsen with disease duration and severity of motor symptoms but remain mild in the first three years after the disease onset [32]. According to the literature, TDPD patients experience lower severity in ANS dysfunction than patients of the postural instability and gait difficulty phenotypes [33,34]. In our study, we did not observe any statistically significant differences in subjective symptoms of ANS dysfunction among the groups. The lack of difference in subjective symptoms in our study was probably related to small groups, short duration of PD, and including only TDPD patients. In a previous study, ET patients exhibited some subjective symptoms of orthostatic intolerance, but objective cardiovascular tests did not indicate dysautonomia [11]. As suggested by the authors of that study, these symptoms could potentially be attributed to other abnormalities, such as vestibular or cerebellar pathology. This may also explain the lack of difference among the groups in subjective symptoms of dysautonomia in our study [11].

ANS functions in ET were also assessed objectively by some studies using medical equipment. The study by Nassar et al. found that ET patients had dysautonomia in a series of tests including heart rate variation during deep breathing, active standing, and the Valsalva maneuver. They also exhibited an abnormal SBP response during active standing and a hand grip test, as well as an abnormal sympathetic skin response. In that study, ANS dysfunction was correlated with disease severity [15]. Habipoglu et al. discovered abnormalities in sympathetic skin response, but no RR interval variation during rest and deep breathing in ET [14]. Another study found that ET patients more frequently had SBP drop ≥20 mmHg and/or DBP drop ≥10 mmHg during the first minute of the tilt test, whereas no difference was observed between the ET and control groups during the 3rd and 5th minutes [35]. However, this observed SBP/DBP drop only in the first minute of the orthostatic test did not meet definitions of classical OH or initial OH [28]. Complex autonomic evaluation of ET patients and controls, including symptoms of orthostatic intolerance, head-up tilt test, supine hypertension, ambulatory blood pressure monitoring, 24 h HRV analysis and cardiac 123I-MIBG-scintigraphy, found only differences in symptoms of orthostatic intolerance. However, there was no significant difference between ET patients with and without orthostatic intolerance symptoms, so it is likely they were not related to autonomic dysfunction [11]. Two previous studies compared HRV in PD and ET patients; both of them found lower HRV in PD patients, and concluded that it could be a potentially useful biomarker to differentiate the diseases [10,12]. A study by Yoon et al. assessed 5 min HRV in 23 ET and 27 early-stage TDPD patients along with 23 healthy controls and found that LF, total spectral power, SDNN, HF, and LF/HF ratio are potentially useful biomarkers for differentiation of TDPD and ET, with best efficacy for LF (AUC = 0.87) [12]. Salsone and colleagues performed HRV analysis of 12 h daytime ECG in ET, PD, and control groups consisting of 10 patients each with diagnoses verified by cardiac 123I-MIBG-scintigraphy and DAT-SPECT. The study found that PD patients had lower HRV compared to ET and controls. All of the HRV parameters were potentially useful in the differentiation of the diseases, but only LF had 100% sensitivity and specificity [10].

In our study we found OH and HRV abnormalities in PD, but not in ET, which is in agreement with all but one of the previous studies that have assessed cardiovascular dysautonomia in ET [10,11,12,14,15,35]. In concordance with the abovementioned studies comparing HRV in PD and ET, in our study, we found that some of the parameters could be potentially useful biomarkers in differential diagnosis, especially LF. Some differences might be associated with less strict inclusion criteria and lack of using cardiac 123I-MIBG- scintigraphy or DAT-SPECT to verify diagnosis for most of the patients in our study, and in the case of study by Salsone, difference in duration of ECG used for HRV analysis [10,12]. Few studies have assessed the presence of OH in ET. Two of them did not find that classically defined OH is more prevalent in ET than in controls, and one described a significant difference in SBP after 3 min of active standing [11,15,28,35]. The study by Nassar et al. found a larger SBP drop in ET patients during active standing [15]. In our study, we used the tilt test. This might contribute to the difference between the study by Nassar et al. and our study because there are some data which have shown some differences between the two approaches [36]. None of these studies compared the presence of OH in TDPD and ET [11,15,35]. In our study, we found that OH is common in TDPD, but not in ET. Moreover, SBP drop during the tilt test might be the next potential biomarker helpful in the differentiation of the diseases. However, this result should be treated with caution because the cut point was in a normal range.

As previously described, dysautonomia has been found in ET patients by a few studies, but the existence of cardiovascular ANS dysfunction is dubious in the disease [9,10,11,12,13,14,15,35]. However, the presence of dysautonomia is well-established in PD and other α-synucleinopathies including multiple system atrophy (MSA), dementia with Lewy bodies, and pure autonomic failure [8]. OH and urinary dysfunction are pivotal features of MSA diagnostic criteria [37]. ANS dysfunction was also described in progressive supranuclear palsy, even if predominant autonomic failure is a mandatory exclusion criterion of PSP [24,38,39]. Dysautonomia in PD includes multiple problems like constipation, dysphagia, genitourinary dysfunction, and thermoregulatory abnormalities [7,8]. Cardiovascular dysautonomia in PD includes supine and nocturnal hypertension, orthostatic and postprandial hypotension, and reduced HRV [7,8,24,40]. Dysautonomia could be present even before the onset of motor symptoms, but its prevalence and severity progresses with the disease’s duration and severity [8,32]. For example, the prevalence of OH is 14% at the beginning of the disease, but 54% in advanced stages [8]. ANS abnormalities, especially cardiovascular problems, are associated with lower quality of life, falls, cognitive impairment, and shorter survival in PD [8,41,42]

There are a few methods helpful in the differentiation of TDPD and ET including DAT-SPECT, 123I-MIBG-scintigraphy, and transcranial sonography [4,5,6]. Transcranial sonography is an inexpensive and widely available method with sensitivity and specificity of 85% and 84%, respectively. However, it is highly dependent on the investigator’s experience and the presence of an appropriate temporal bone window [5]. On the other hand, DAT-SPECT and 123I-MIBG-scintigraphy are relatively expensive and not widely available. According to our study and two previously mentioned studies, HRV analysis is a cost-effective, highly available, and easy to use method, which could be helpful in the differential diagnosis of the diseases [10,12]. Similarly, the assessment of SBP drop during the tilt test could provide benefits, but in this case, our results should be treated with much caution. The proper differential diagnosis of both diseases might be challenging [3]. HRV analysis and tilt test after replication and validation in a larger group of patients could significantly improve diagnostics of both diseases, especially in dubious cases which can not be assessed using other methods. Differential diagnosis is important because there are significant differences in treatment and prognosis between the diseases. These diagnostic factors also might be useful in future scientific studies to improve the differentiation of the diseases.

Our study had some limitations. The first was a small number of patients, although it was comparable to other studies addressing the subject of ANS dysfunction as a potential biomarker in the differential diagnosis of ET and PD [10,12]. Elderly patients with ET and PD frequently have comorbidities demanding treatment, which makes it difficult to find a large group of subjects without any potential confounders and thus limits the number of included patients. Effects of dopaminergic and other drugs and comorbidities should be considered as the factors potentially affecting results.

Secondly, patients did not have histopathological confirmation of their diagnosis and in most cases, 123I-MIBG-scintigraphy or DAT-SPECT were not performed.

In our study, there was longer disease duration in ET patients in comparison to TDPD patients, which might potentially impact results. However, the presence of cardiovascular dysautonomia in ET is dubious in the context of most previous studies and there is no strong evidence that dysautonomia is associated with longer ET duration, so it is likely that the longer ET duration in the studied patients did not affect the results [10,11,12,15,35]. On the other hand, longer duration of PD is related to more severe dysautonomia [8,32]. Differential diagnosis of TDPD and ET is usually problematic at the beginning of the diseases. The group consisted of TDPD patients with relatively short disease duration, when autonomic dysfunction is less severe, and this seemed to be beneficial in the clinical context of the study.

This is a small cross-sectional study and did not explore potential sex differences, nor assess dysautonomia in different age groups. In some studies, dysautonomia was more severe in male PD patients [41,43]. Additionally, HRV in healthy adults typically decreases with age, and it is lower in healthy females [44]. There were no significant sex and age differences in the study between groups, but some effects of age and sex might be present and affect the study results.

## 5. Conclusions

Our study revealed that cardiovascular dysautonomia is present in TDPD, but is absent in ET. The study results show the usefulness of SBP drop during tilt test in the differentiation of ET and TDPD, but this result should be treated with strong caution due to the small number of patients and the cut point being located in the normal range. Moreover, our study is one of few which indicate that HRV could be an inexpensive, widely available, and helpful biomarker in the differentiation of ET and TDPD, even in the presence of some confounders, which are common in these groups of patients. Large studies are needed to confirm the usefulness of HRV analysis and SBP drop in differentiation of the diseases, as well as to determine subgroups of ET and TDPD patients who could take advantage of these tools. The potential effects of age and sex on ANS in PD and ET also should be explored.

## Figures and Tables

**Table 1:** Demographic data, comorbidities, and medications.

	ET	TDPD	Control Group	*p*-Value
**Demographic data and comorbidities**				
Age (years)	67 (60–75)	65 (52–71)	61.5 (56.5–67)	0.220
Sex (% male)	58%	80%	40%	0.060
Disease duration (years)	6 (5–13)	3 (1.5–7)	-	0.015 *
HYs OFF	-	2 (1–2)	-	-
MDS-UPDRS-3 ON (pts)	-	14 (8–27)	-	-
MDS-UPDRS-3 OFF (pts)	-	37.1 ± 19.9	-	-
TETRAS (pts)	22.9 ± 7.9	-	-	-
Arterial hypertension	63%	40%	35%	0.179
Diabetes mellitus	5%	13%	5%	0.587
Heart diseases	26%	0%	10%	0.067
History of cancer ^†^	5%	0%	15%	0.222
Depression	31%	13%	-	0.260
**Medications**				
LEDD (mg)	0 (0–280)	460 (150–750)	0 (0-0)	0.001 *
	Post-hoc analysis: TDPD > ET and control group			
Rasagiline/selegiline	11%	27%	0%	0.045 *
	Post-hoc analysis: not significant			
Amantadine	0%	13%	0%	0.067
Primidone	26%	0%	0%	0.006 *
	Post-hoc analysis: not significant			
α–adrenergic antagonists	21%	20%	0%	0.094
β–blockers	53%	27%	20%	0.079
RAS	42%	33%	5%	0.023 *
	Post-hoc analysis: ET > control group			
Diuretics	11%	7%	0%	0.349
Calcium blockers	26%	20%	10%	0.412
SSRI	16%	20%	0%	0.127
Other antidepressive	5%	7%	0%	0.530
Atypical antipsychotics	0%	7%	0%	0.266

^†^—In all cases patients were after effective treatment of cancer. *—statistically significant differences. Abbreviations: HYs—Hoehn-Yahr scale, TETRAS—tremor research group essential tremor rating assessment scale performance subscale, LEDD—daily levodopa equivalent dose, RAS—renin-angiotensin system antagonists, SSRI—serotonin reuptake inhibitors.

**Table 2:** Autonomic nervous system assessment.

	ET	TDPD	Control Group	*p*-Value
**SCOPA-AUT questionnaire**				
Sum of points in SCOPA-AUT	9 (6–13)	8 (4–17)	5 (3–11)	0.195
Sum of points in SCOPA-AUT without sexual domain	9 (5–11)	6 (3–15)	5 (3–9.5)	0.198
Gastrointestinal domain	1 (1–2)	2 (0–4)	1 (0–1)	0.129
Urinary domain	4 (2–6)	3 (1–5)	2.5 (1–4.5)	0.448
Cardiovasculardomain	0 (0–2)	0 (0–1)	0 (0-0)	0.140
Thermoregulatorydomain	2 (1–3)	1 (0–3)	1 (0–2)	0.457
Pupillomotor domain	1 (0–1)	0 (0–1)	0 (0–1)	0.037 *
	Post-hoc analysis: not significant			
Sexually active	58%	53%	75%	0.359
Sexual domain	1 (0–2)	1 (0–2)	0 (0–1)	0.163
**HRV Analysis ^†^**				
Mean heart rate (bpm)	61.0 ± 8.2	66.1 ± 8.7	63.8 ± 7.2	0.190
SDNN (ms)	32.0 ± 12.0	21.3 ± 7.5	37.2 ± 10.0	<0.001 *
	Post hoc analysis: TDPD < control group and ET			
RMSSD (ms)	21.5 (15.0–25.5)	13.1 (9.2–17.0)	17.8 (15.1–22.1)	0.051
VLF (ms^2^)	381.4 (219.9–768.5)	207.4 (89.6–324.2)	609.3 (450.6–957.2)	<0.001 *
	Post-hoc analysis: TDPD < control group			
LF (ms^2^)	191.9 (133.7–282.5)	83.1 (32.9–116.1)	231.6 (132.3–365.5)	<0.001 *
	Post-hoc analysis: TDPD < control group and ET			
HF (ms^2^)	124.2 (80.7–279.4)	52.5 (26.0–139.3)	104.4 (73.4–193.0)	0.103
LF/HF ratio	1.1 (0.7–2.6)	0.8 (0.4–1.1)	1.7 (1.2–3.5)	0.128
**Orthostatic hypotension assessment**				
ΔSBP [mmHg]	0.6 ± 6.1	14.3 ± 14.6	−0.7 ± 9.2	<0.001 *
	Post-hoc analysis: TDPD > control group and ET			
ΔDBP [mmHg]	−3.9 ± 4.9	0.7 ± 6.7	−3.4 ± 6.1	0.057
ΔHR [bpm]	8.4 (6.6–11.1)	8.1 (4.2–12.5)	8.6 (7.2–13.1)	0.672
SBP ≥ 20 mmHg	0%	33%	0%	<0.001 *
	Post-hoc analysis: TDPD > control group and ET			
DBP ≥ 10 mmHg	0%	13%	5%	0.239
SBP ≥ 20 mmHg and DBP ≥ 10 mmHg	0%	13%	0%	0.067
OH	0%	33%	5%	0.005 *
	Post-hoc analysis: TDPD > ET			
NOH	0%	33%	0%	<0.001 *
	Post-hoc analysis: TDPD > control group and ET			

^†^—Two TDPD and one ET patients did not have HRV analysis due to a temporal unavailability of Biopac system. *—statistically significant results. Abbreviations: TDPD—tremor dominant Parkinson’s disease, ET—essential tremor, HRV—heart rate variability, SDNN—standard deviation of all normal RR intervals RMSSD—root mean square of the successive differences between adjacent normal RR intervals, HF high-frequency band, LF—low-frequency band, VLF—very-low-frequency band, SBP—systolic blood pressure, DBP—diastolic blood pressure, ∆SBP—systolic blood pressure drop, ∆DBP—diastolic blood pressure drop, OH—orthostatic hypotension, NOH—neurogenic orthostatic hypotension.

## Data Availability

The data presented in this study are available on request from the corresponding author (privacy restrictions).
